# Supplementary material for: Nanocomposites Based on Spin-Crossover Nanoparticles and Silica-Coated Gold Nanorods: A Nonlinear Optical Study
Source: Molecules. 2023 May 19;28(10):4200. doi: 10.3390/molecules28104200 (PMC10222092; doi:10.3390/molecules28104200)
Supplement: Supplementary file 1 [file molecules-28-04200-s001.zip › molecules-2404274-supplementary.pdf]

Nanocomposites based on Spin Crossover Nanoparticles and Silica Coated Gold Nanorods: A Nonlinear Optical Study

Eleni Zygouri [1], Aristeidis Stathis [2,3] Stelios Couris [2,3]*, Vassilis Tangoulis[1]*

[1] Laboratory of Inorganic Chemistry, Department of Chemistry, University of Patras, 26504 Patras, Greece; eleni0503zig@hotmail.com
[2] Department of Physics, University of Patras, 26504 Patras, Greece; a.stathis@iceht.forth.gr
[3] Institute of Chemical Engineering Sciences (ICE-HT), Foundation for Research and Technology-Hellas (FORTH), 26504 Patras, Greece

* Correspondence: couris@upatras.gr (S.C.); vtango@upatras.gr (V.T.)

Supplementary material

INDEX

Figure S1. Gaussian Distributions of Sizes of AuNRs ____________________ *3*

*S1. Nonlinear Optical Measurements*____________________ *3*

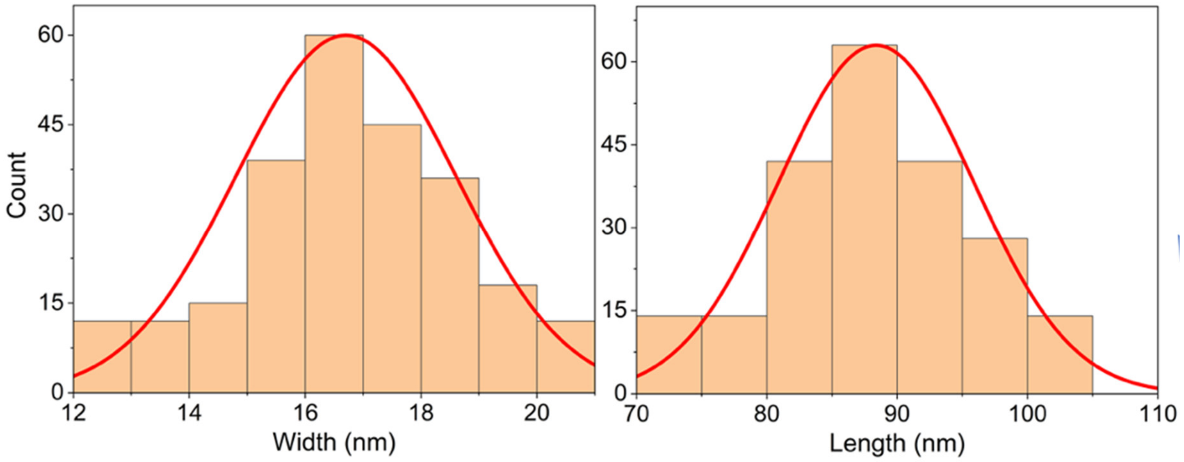

Figure S1. Gaussian Distributions of Sizes of AuNRs

S1. Nonlinear Optical Measurements

The nonlinear optical response of the prepared samples was studied by means of the Z-scan technique [39]. Z-scan is a relatively simple, single beam, experimental technique which allows for the simultaneous determination of the nonlinear absorption (i.e., the nonlinear absorption coefficient β) and the nonlinear refraction (i.e., the nonlinear refractive index parameter γ') of a sample. The technique is based on the measurement of the transmittance of a sample, as it moves along the propagation axis of a focused laser beam, i.e., the z-axis, thus experiencing different laser intensity at each position. A schematic of a typical Z-scan experimental setup is presented in Figure S2.

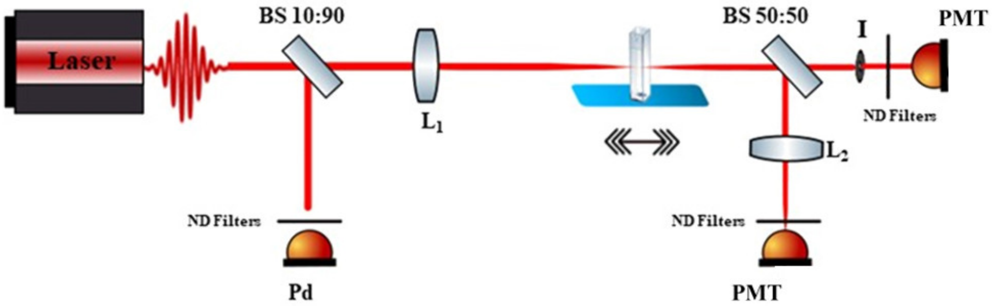

Figure S2. Schematic of the Z-scan experimental setup (Laser: laser beam, BS: Beam Splitter, Pd: Photodiode, L: lens, ND Filters: neutral density filter, I: aperture, PMT: photomultiplier).

As the sample moves towards the focal plane of the laser beam, focused by lens 1 (L_1), it experiences gradually higher laser intensities giving rise to nonlinear absorption and refraction, which result in modification of the transmission characteristics of the sample. The modifications of the transmittance characteristics are then monitored by splitting the transmitted through the sample laser beam and measuring it by two different experimental configurations, namely, the "Open Aperture" (OA) Z-scan and the "Closed Aperture" (CA) Z-scan. In the former transmission measurement, the transmitted laser beam is totally collected (e.g., by lens L_2) and is measured by means of a photomultiplier (PMT), while in the latter case, the transmitted laser beam

after having passed through a small aperture (I) positioned in the far field of the focusing lens L_1, is also measured by a PMT. To eliminate any noise in the recorded signal arising from possible laser power fluctuations that may occur during the scan, a reference detector (Pd) is used to monitor and normalize the transmittance. An OA Z-scan recording can present a transmission maximum or minimum. The presence of a transmission maximum indicates a negative sign nonlinear absorption coefficient β ($\beta < 0$), corresponding to saturable absorption (SA) behavior, while the presence of a transmission minimum indicates a positive sign β ($\beta > 0$), corresponding to reverse saturable absorption (RSA) behavior. According to the Z-scan formalism, the OA transmission recording can be described by the following equation (S1):

$$\mathrm{T}(z) = \frac{1}{\sqrt{\pi}\left[\frac{\beta I_0 L_{eff}}{\left(1+\frac{z^2}{z_0^2}\right)}\right]}\int_{-\infty}^{+\infty} n\left[1+\frac{\beta I_0 L_{eff}}{\left(1+\frac{z^2}{z_0^2}\right)}e^{-t^2}\right]dt \qquad \text{(S1)}$$

where β is the nonlinear absorption coefficient, I_0 is the on-axis peak irradiance, z_0 is the confocal parameter (or Rayleigh length), and L_{eff} is given by the expression: $L_{eff} = \frac{[1-e^{-\alpha_0 L}]}{\alpha_0}$ with α_0 being the linear absorption coefficient at the laser excitation wavelength and L is the interaction length of the laser beam with the material, i.e., the sample thickness. By fitting the OA Z-scan recording with equation (1) the nonlinear absorption coefficient β can be deduced. Then, the imaginary part, i.e., $Im\chi^{(3)}$, of the third-order nonlinear susceptibility $\chi^{(3)}$, can be calculated from the following equation (S2):

$$Im\chi^{(3)}(esu) = 10^{-7}\frac{c^2 n_0^2}{96\pi^2\omega}\beta\left(\frac{cm}{W}\right) \qquad \text{(S2)}$$

where c is the speed of light (in cm s^{-1}), n_0 is the refractive index and ω is the frequency of the incident beam (in s^{-1}).

In the case of CA Z-scan recording, its shape can exhibit a pre-focal transmission minimum (valley) followed by a post-focal transmission maximum (peak) or a pre-focal transmission maximum (peak) followed by a post-focal transmission minimum (valley), indicating positive or negative NLO refractive index parameter γ', corresponding to self-focusing or self-defocusing, respectively. By fitting the CA Z-scan recording by equation (S3), the nonlinear refractive index parameter γ' can be determined:

$$T(z) = 1 + 4\gamma' k_0 L_{eff}\frac{x}{(1+x^2)(9+x^2)} \qquad \text{(S3)}$$

where $x = \frac{z}{z_0}$, $k_0 = \frac{2\pi}{\lambda}$ is the wavevector and λ is the laser wavelength, all in free space. The quantities z_0 and L_{eff} have been defined previously.

From the so determined nonlinear refractive index parameter γ', the real part, $Re\chi^{(3)}$, of the third-order nonlinear susceptibility $\chi^{(3)}$, can be calculated using the following equation (S4):

$$Re\chi^{(3)}(esu) = 10^{-6}\frac{cn_0^2}{480\pi^2}\gamma'\left(\frac{cm^2}{W}\right) \qquad \text{(S4)}$$

As the nonlinear absorption coefficient β is related to the $Im\chi^{(3)}$, and the nonlinear refractive index parameter γ' is related to the $Re\chi^{(3)}$, then the magnitude of the third-order nonlinear susceptibility $\chi^{(3)}$ of the sample can be obtained using the following relation:

$$\chi^{(3)} = \sqrt{(Im\chi^{(3)})^2 + (Re\chi^{(3)})^2} \qquad \text{(S5)}$$

For the nonlinear optical studies, a 4 ns Q-switched Nd: YAG laser (EKSPLA, NT 342/3/UVE/AW Series, Vilnious, Lithuania), operating at a repetition rate of 1-10 Hz, has been used. Z-scan measurements were performed at both 1064 and 532 nm (i.e., the fundamental and the second harmonic output of the laser, respectively), to achieve resonant excitation conditions of the transverse and longitudinal Surface Plasmon Resonances (SPRs) of the AuNRs@SCO hybrids. By exciting the SPRs resonantly, the maximum photo-thermal heating can be achieved. The laser beam was focused into the sample by means of 20 cm focal-length quartz plano-convex lens, while its energy was monitored by means of a calibrated joulemeter (Coherent-EnergyMax

J-10MT-10kHZ, Coherent Inc., Santa Clara, CA, USA). The diameter of the laser beam at the focal plane was measured at both excitation wavelengths using a CDD camera. It was found to be 18 and 30 μm at 532 and 1064 nm, respectively.
